# Anti-*Candida albicans* Effects and Mechanisms of Theasaponin E1 and Assamsaponin A

**DOI:** 10.3390/ijms24119350

**Published:** 2023-05-27

**Authors:** Yuhong Chen, Ying Gao, Mingan Yuan, Zhaisheng Zheng, Junfeng Yin

**Affiliations:** 1 Key Laboratory of Tea Biology and Resources Utilization, Tea Research Institute of Chinese Academy of Agricultural Sciences, Ministry of Agriculture, 9 South Meiling Road, Hangzhou 310008, China; chenyuhong@tricaas.com; 2 Graduate School of Chinese Academy of Agricultural Sciences, Beijing 100081, China; 3 Jinhua Academy of Agricultural Science, Jinhua 321000, China; minganyuan@126.com (M.Y.); zzs165@163.com (Z.Z.)

**Keywords:** *Camellia sinensis* seeds, theasaponin E1, assamsaponin A, antifungal activity, transcriptome

## Abstract

*Candida albicans* is an opportunistic human fungal pathogen, and its drug resistance is becoming a serious problem. *Camellia sinensis* seed saponins showed inhibitory effects on resistant *Candida albicans* strains, but the active components and mechanisms are unclear. In this study, the effects and mechanisms of two *Camellia sinensis* seed saponin monomers, theasaponin E1 (TE1) and assamsaponin A (ASA), on a resistant *Candida albicans* strain (ATCC 10231) were explored. The minimum inhibitory concentration and minimum fungicidal concentration of TE1 and ASA were equivalent. The time–kill curves showed that the fungicidal efficiency of ASA was higher than that of TE1. TE1 and ASA significantly increased the cell membrane permeability and disrupted the cell membrane integrity of *C. albicans* cells, probably by interacting with membrane-bound sterols. Moreover, TE1 and ASA induced the accumulation of intracellular ROS and decreased the mitochondrial membrane potential. Transcriptome and qRT-PCR analyses revealed that the differentially expressed genes were concentrated in the cell wall, plasma membrane, glycolysis, and ergosterol synthesis pathways. In conclusion, the antifungal mechanisms of TE1 and ASA included the interference with the biosynthesis of ergosterol in fungal cell membranes, damage to the mitochondria, and the regulation of energy metabolism and lipid metabolism. Tea seed saponins have the potential to be novel anti-*Candida albicans* agents.

## 1. Introduction

*Candida albicans* is one of the ubiquitous commensal fungi and the most common causative pathogen found on the skin and mucous membranes such as the oral cavity, gastrointestinal tract, and vagina [1,2,3]. It grows as a yeast, pseudohyphae, or true hyphae depending on environmental conditions. *C. albicans* is more infectious when transformed from yeast form to hypha form [4,5]. According to the data on fungal infections in hospitals, the mortality rate of immunocompromised patients infected with *C. albicans* is estimated to be 40%, making *C. albicans* the most serious fungal invasive infectious fungal strain [6]. *C. albicans* has expanded from simple endogenous infections in hospitals to exogenous infections that may be acquired through the hands of healthcare workers, contaminated fluids and biomaterials, and inanimate environments [7]. The disinfection and sterilization of *Candida albicans* on equipment and the environment is also a potential way to prevent and control the spread of intervention. Currently, there are five common drugs used in clinical applications for the treatment of *C. albicans* infections, which are classified as azoles, polyenes, allylamines, candins, and flucytosine according to their sites of action [8]. However, more and more antibiotic-resistant strains are appearing, leading to an urgent need to explore novel antifungal drugs which are efficient, safe, and cost-effective.

Natural products are popular sources for screening antifungal drugs. Tea saponins are triterpenoids with various physiological functions such as anti-inflammatory, antiviral, anticancer, and antifungal activities, as well as gastrointestinal protection [9,10,11,12,13,14,15,16]. Previous studies have shown that tea saponins exhibited good inhibitory activity against the skin-pathogenic fungus *Microsporum audouinii* [17]. They also had an inhibitory effect on *Botrytis cinerea*, which was the main cause of the rot and mold of nectarines [15]. In particular, tea saponins had inhibitory activity against *C. albicans* [16,18]. Zhang et al. found that after treatment with tea saponins, the morphology of *C. albicans* was changed, including a shrinkage in appearance and a rupture of the cell wall and plasma membrane [16]. Li et al. explained that the induction of oxidative stress might be an important antifungal mechanism of saponins because increases in intracellular ROS and mitochondrial dysfunction were observed in *C. albicans* [18]. However, tea saponins are composed of a series of saponins with similar structures. According to differences in sapogenin, glycone, and organic acids, more than 40 monomers have been identified in *Camellia sinensis* seeds [19]. The active components in tea seed saponin mixtures and their antifungal mechanisms against *C. albicans* are still to be elucidated.

**Figure 1:**
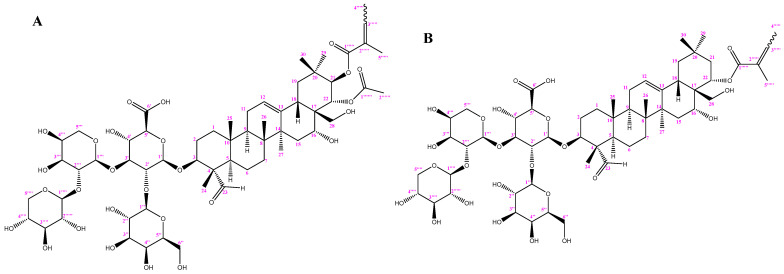
The structures of theasaponin E1 (**A**) and assamsaponin A (**B**).

In this study, *C. albicans* ATCC 10231, which is resistant to most antifungal drugs (fluconazole, anidulafungin, itraconazole, and voriconazole), was used as the experiment strain, and the anti-*C. albicans* activities and mechanisms of two tea seed saponin monomers, theasaponin E1 (TE1) and assamsaponin A (ASA) (Figure 1), were evaluated against *C. albicans* ATCC 10231 through physiological and biochemical examinations, morphological characteristics, and transcriptome analysis. These findings will provide a theoretical basis for the development and utilization of tea seed saponins.

## 2. Results

### 2.1. Antifungal Activities of TE1 and ASA

**Figure 2:**
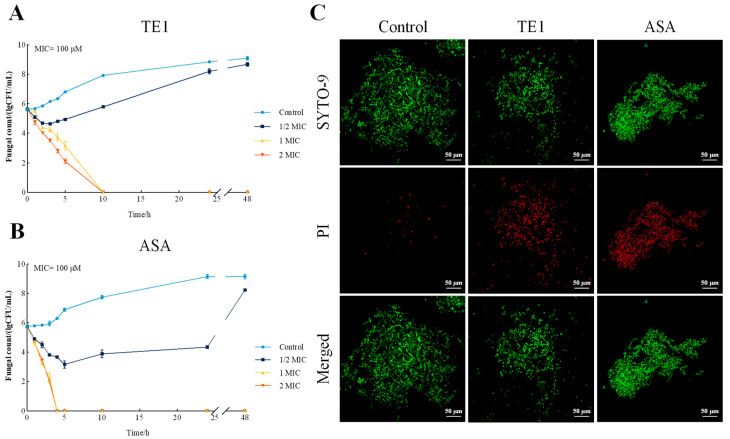
Antifungal activities of theasaponin E1 (TE1) and assamsaponin A (ASA). (**A**) Time-kill curves of TE1; (**B**) Time-kill curves of ASA; (**C**) Viability fluorescent staining of *C. albicans* 10231 observed by CLSM. Data are presented as (means ± SD) of three replicates.

The minimum inhibitory concentrations (MICs) were determined using the CLSI broth microdilution method (Table 1). TE1 showed an obvious fungicidal activity effect on the fluconazole-resistant *C. albicans* strain (ATCC 10231), with an MIC and minimum fungicidal concentration (MFC) of 100 µM and 100 µM, respectively, representing an MFC/MIC ratio of 1 [20]. ASA showed the same effect. Time–kill curves (Figure 2A,B) showed that TE1 and ASA exhibited a good and rapid fungicidal effect within 4–10 h. At 1 MIC of TE1, the cell count of *C. albican* was reduced from 5.64 to 4.02 log CFU/mL after 2 h, and finally to 0 log CFU/mL after 10 h. At 1 MIC of ASA, the cell count of *C. albican* was reduced from 5.76 to 3.21 log CFU/mL after 2 h, and finally to 0 log CFU/mL after 4 h. This implies that ASA had a better fungicidal efficiency rate than TE1. Moreover, STYO-9/propidium iodide (PI) staining was used to measure the cell damage (Figure 2C). SYTO-9 stains all microbes and displays green fluorescence, whereas PI merely stains microbes with compromised membranes and displays red fluorescence. Compared with the control group, the red fluorescence intensities (dead cells) of *C. albicans* treated with TE1 and ASA for 2 h were significantly increased (Figure 2C). The above results indicate that TE1 and ASA exhibited high antifungal activities against *C. albicans*.

### 2.2. Effects of TE1 and ASA on the Cell Wall and Membrane of C. albicans

**Figure 3:**
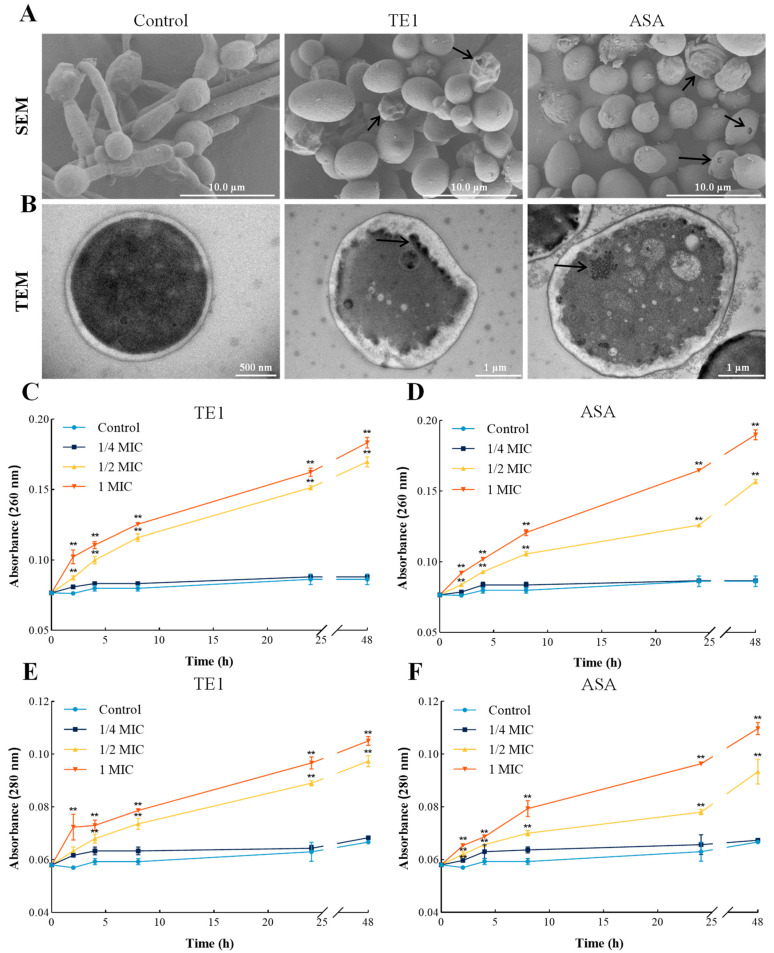
Theasaponin E1 (TE1) and assamsaponin A (ASA) destroyed the structure of the *C. albicans* 10231 cell wall and membrane. (**A**) Representative SEM images of *C. albicans* treated with 1 MIC of TE1 and ASA. Cells with indentations and breakages (arrows) can be seen. (**B**) Representative TEM images of *C. albicans* treated with 1 MIC of TE1 and ASA. TE1- or ASA-treated cells in which aggregations of tiny vacuoles are seen (arrows). (**C**,**D**) The content of nucleic acids in *C. albicans* culture medium quantified after treatment with different concentrations of TE1 and ASA. (**E**,**F**) The content of protein in *C. albicans* culture medium quantified after treatment with different concentrations of TE1 and ASA. Data are presented as (means ± SD) of three replicates. ** *p* < 0.01 were obtained for treated samples vs. control.

Previous studies have demonstrated that many antibiotics display antifungal activity by impairing cell membrane integrity and permeability [21]. Scanning electron microscopy (SEM) showed that cells cultured in fresh culture medium formed a mycelium form with smooth cell walls, while cells cultured in TE1- or ASA-containing medium maintained yeast morphology and exhibited several morphological changes, such as shrinkage, indentation, and breakage (Figure 3A). As shown in Figure 3B, changes in the ultrastructure of *C. albicans* cells with a 2 h TE1 or ASA treatment were observed by transmission electron microscope (TEM). The cell walls were not clearly discernible, and many small vacuoles gathered near the cell membrane in TE1- or ASA-treated cells, indicating the loss of membrane integrity. The integrity of cell membranes was further evaluated by measuring the leakage of cellular contents such as nucleic acids and proteins. The OD260 and OD280 values of the culture medium represented the concentrations of extracellular nucleic acids and proteins, respectively. The leakage of nucleic acids (Figure 3C,D) and proteins (Figure 3E,F) was increased by TE1 and ASA in a dose- and time-dependent manner. The OD260 values of the culture medium from *C. albicans* treated with 1 MIC TE1 and ASA for 48 h were 2.12 and 2.20 times higher than the control, respectively. PI is a dye that penetrates the cell membrane of damaged cells and combines with the DNA of the nucleus to generate a red fluorescent PI complex, thereby being used to verify the integrity of the cell membrane [22]. The higher red fluorescence intensity of TE1- or ASA-treated cells provided more evidence of impaired membrane integrity and permeability (Figure 2C). The above results indicate that TE1 and ASA destroyed the structure of the fungal cell walls and membranes.

Sorbitol is used as an osmotic stabilizer of fungal growth. Cell walls cannot grow or be repaired without sorbitol [23,24]. To investigate the role of impairing cell walls in the TE1- and ASA-induced inhibition of *C. albicans*, *C. albicans* was cultured in the medium with or without sorbitol. As shown in Table 2, the MIC values of TE1 and ASA did not increase within 7 days, suggesting that impairing cell walls might not be vital for the TE1- and ASA-induced inhibition of *C. albicans*. On the contrary, the MIC of caspofungin (CPF), a medication known to display antifungal activity using sorbitol, was increased from 0.25 to 8 µg/mL.

Most of the previous literature has shown that the fungicidal activity of saponins is attributed to their interactions with sterol [25,26]. To verify whether TE1 and ASA had similar functions, different concentrations of ergosterol were added to observe their effects on the MICs of TE1 and ASA. The results (Table 3) showed after adding 250 µg/mL of ergosterol, the MICs of TE1 and ASA were increased 2-fold, while the MIC of amphotericin B (AMB, known to bind to ergosterol) was increased 64-fold. This indicates that the damage to the cell membrane by binding ergosterol might be involved in the TE1- and ASA-induced inhibition of *C. albicans*.

### 2.3. Effect of TE1 and ASA on Intracellular Reactive Oxygen Species (ROS) and Mitochondria

**Figure 4:**
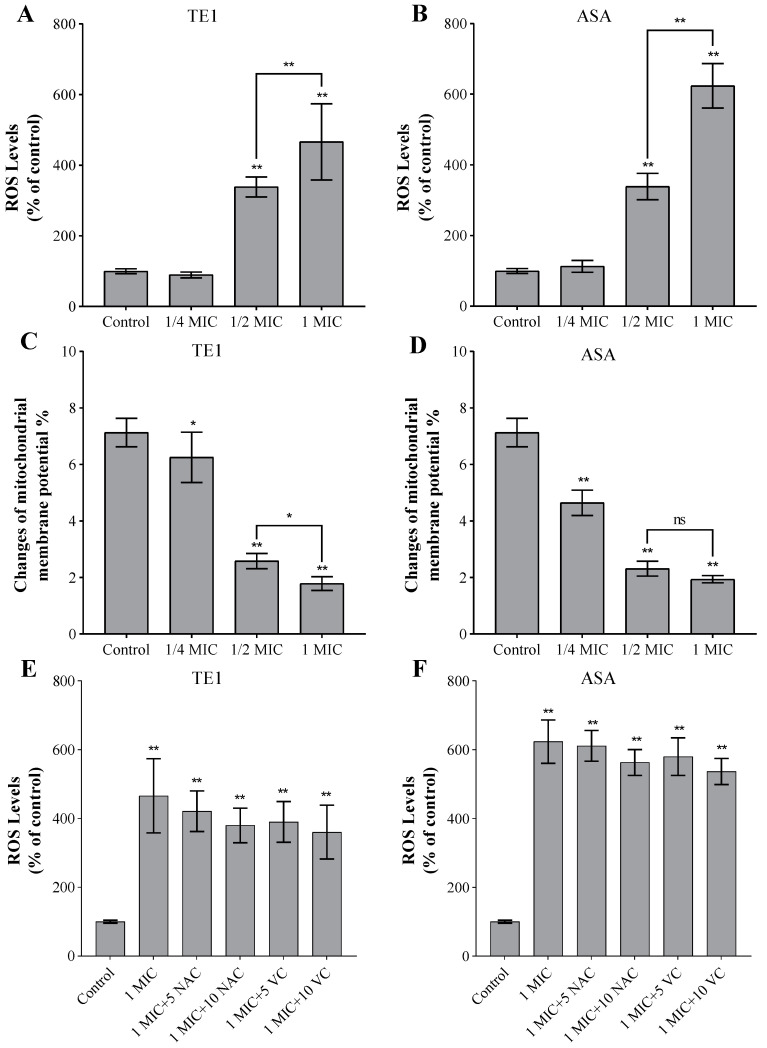
Effects of theasaponin E1 (TE1) and assamsaponin A (ASA) on the reactive oxygen species (ROS) production and mitochondrial membrane potentials (MMP) in *C. albicans* 10231. (**A**,**B**) The production of ROS from *C. albicans* treated with different concentrations of TE1 and ASA was determined by 2′,7′-Dichlorodihydrofluorescein diacetate (DCFH-DA); (**C**,**D**) MMP of *C. albicans* treated with various concentrations of TE1 and ASA were detected by JC-1 fluorescence. (**E**,**F**) The production of ROS from *C. albicans* treated with 1 MIC of TE1 and ASA following the addition of different concentrations (5 mM and 10 mM) of antioxidant (N-acetyl-L-cysteine, NAC or vitamin C, VC). Data are means (±SD) of three replicates. * *p* < 0.05 or ** *p* < 0.01 were obtained for treated samples vs. control, and ns indicated not significant.

Mitochondria are essential for the initiation of intrinsic apoptosis, which is triggered by an increase in ROS. According to Figure 4A,B, ROS were generated in *C. albicans* cells in a concentration-dependent manner after TE1 and ASA treatment, respectively, suggesting that TE1 and ASA caused ROS accumulation in *C. albicans*. The JC-1 staining assay was performed to examine the effect of TE1 and ASA on the mitochondria membrane potential (MMP) (Figure 4C,D). Both compounds dose-dependently decreased MMP. A 1/4 MIC of TE1 reduced the accumulation of fluorescence by 12.33%, while a 1/4 MIC of ASA reduced it by 34.83%. There was no significant change in the fluorescence ratio between cells treated with 1/2 MIC and 1 MIC ASA (*p* > 0.05), both of which had a significant impact on the mitochondria. According to the results, TE1 and ASA induced the intracellular accumulation of ROS and a decrease in MMP, which could result in the apoptosis of *C. albicans*.

To investigate the role of ROS in the inhibitory effect of TE1 and ASA on *C. albicans*, the intracellular ROS level of *C. albicans* after treatment with N-acetyl-L-cysteine (NAC) or vitamin C (VC) was compared with that of untreated control cells (Figure 4E,F). However, the addition of antioxidants (NAC or VC) did not weaken the inhibitory effect of TE1 and ASA and could not prevent the increase in the intracellular ROS level. From these results, it was speculated that the two saponins caused oxidative damage to *C. albicans*, but it might not be the main cause of TE1- or ASA-induced cell death.

### 2.4. Transcriptomic Analysis of C. albicans with TE1 and ASA Treatment

To gain insights into the possible molecular mechanism in the antifungal activity of TE1 and ASA, transcriptome analyses by RNA sequencing (RNA-seq) were performed. The Q20 of each piece of raw data was more than 97%, which indicated its accuracy and reliability and that it was worthy of further analyses (Table S1). A total of 2135 DEGs were identified between the control samples and the TE1-treated samples, including 1344 upregulated and 791 downregulated genes (Table S2). A total of 2105 DEGs were identified between the control samples and the ASA-treated samples, including 1339 upregulated and 766 downregulated genes (Table S2). In addition, only 17 DEGs were identified between the TE1-treated samples and the ASA-treated samples, which suggested that TE1 and ASA had similar fungicidal mechanisms.

**Figure 5:**
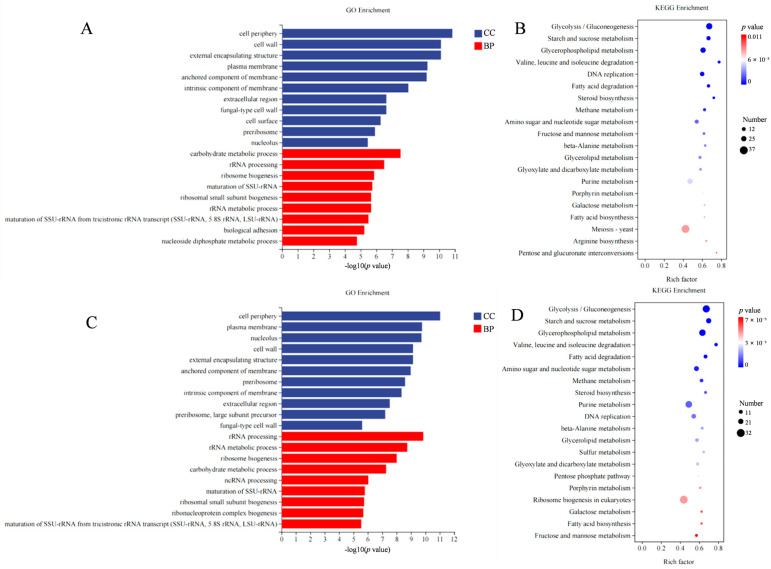
Transcriptome analysis of the effects of theasaponin E1 (TE1) and assamsaponin A (ASA) on *C. albicans* 10231. (**A**,**C**) DEGs of the top 20 enriched GO terms of TE1 and ASA; (**B**,**D**) KEGG enrichment analysis of DEGs of TE1 and ASA.

Gene ontology (GO) analysis showed that the DEGs between the control samples and TE1- or ASA-treated samples mainly included two categories, which were the cellular component (CC) comprising “cell periphery”, “cell wall”, and “plasma membrane”, and the biological process (BP), comprising “carbohydrate metabolic process”, “rRNA processing”, and “ribosome biogenesis” (Figure 5A,C). These results indicate that TE1 and ASA damage cell membrane function. The Kyoto Encyclopedia of Genes and Genomes (KEGG) database analysis revealed that most DEGs between the control samples and the TE1- or ASA-treated samples were related to “glycolysis/gluconeogenesis”, “starch and sucrose metabolism”, “glycerophospholipid metabolism”, “fatty acid degradation”, “steroid biosynthesis”, and “DNA replication”, which could be further categorized into three major pathways: carbohydrate metabolism, lipid metabolism, and replication and repair (Figure 5B,D).

**Figure 6:**
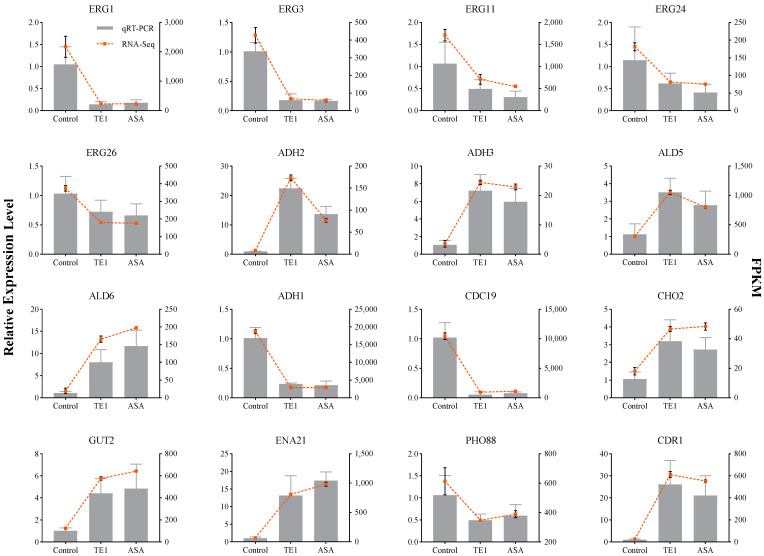
qRT-PCR validation of 16 DEGs. Data are means (±SD) of three replicates. FPKM: fragments per kilobase of exon model per million mapped fragments.

Quantitative real-time PCR (qRT-PCR) was used to confirm the changes in the 16 DEGs identified by RNA-seq, including genes related to glucose metabolism, ergosterol biosynthesis, fatty acid degradation, and membranes. The qRT-PCR results were consistent with the gene expression trends observed by transcriptome sequencing (Figure 6).

## 3. Discussion

With the increase in drug resistance in fungi, natural products are viewed as a potential alternative for controlling infectious diseases. Azole drugs are common low-toxicity antifungal drugs, but due to long-term use, some resistant strains have emerged. The biochemical basis for the development of resistance to azoles includes four aspects. First, mutations in the 14α-demethylase, which serves as the target of the action, result in a decreased affinity between azoles and the enzyme. Second, lesions in the Δ 5(6)-desaturase result in the production of 14α-methyl fecosterol instead of ergosterol. Third, the overexpression of 14α-demethylase, which attenuates the azoles-induced decrease in ergosterol enhances drug resistance [27]. The changes in these enzymes themselves may all come from mutations in the *ERG11* gene. Fourth, the overexpression of efflux pump genes leads to a decrease in the antifungal drug concentration inside the fungi and results in drug resistance. Discovering novel targets of action, inhibiting glucan synthase enzyme activities, and modifying compound configurations to enhance affinity with enzymes are possible ways to overcome and solve the issue. VT1129 and VT1161 are triazole compounds whose affinity to the Candida cytochrome P-450 enzyme (CYP51) is at least 1000 times higher than that of homologous zymosomes in the human body [28]. SCY-078 affects the surface of β-(1,3)-D-glucan synthase and achieves antifungal effects by destroying cellular walls [29,30]. T-2307 disrupts fungal mitochondrial membranes, thereby interfering with cell energy metabolism [31]. The antifungal activity of saponins is comparable to or even stronger than traditional antifungal drugs, and some saponins inhibit drug-resistant strains [32,33]. Yin et al. found that analogues of *Panax stipulcanatus* saponins have good antifungal activity against *C. albicans* and have synergistic antifungal effects against fluconazole-resistant strains [34]. Coleman et al. also found saponins against the resistant strains at low concentrations (16 and 32 µg/mL) [33]. Here, we found similar results. *C. albicans* ATCC 10231 was resistant to fluconazole, while the saponin mixture and saponin monomers (TE1 and ASA) extracted from tea seeds have antifungal effects towards it. Additionally, saponins have relatively low cytotoxicity to animal hosts [33,35]. Their high effectiveness and safety make saponins ideal alternative antifungal agents.

*C. albicans*, one of the most common human fungal pathogens [36], has emerged as one of the major problems for nosocomial infections, posing a fatal factor for patients with septic shock [37,38]. Triterpenoid saponins have been reported to inhibit the proliferation of various *Candida* species [39,40,41]. Tea saponins, which belong to triterpenoid saponins, are abundant in tea seeds and have an inhibitory effect against *C. albicans* [16,18]. In this study, two tea seed saponin monomers, TE1 and ASA were prepared from a high-purity tea seed saponin mixture. The saponin mixture (purity > 96%) along with TE1 and ASA, had inhibitory activity against *C. albicans* ATCC 10231. They all showed high antifungal efficacy within 2 h. When treated for 10 h, none of the *C. albicans* were alive. The MIC of TE1 was 100 μM (123 µg/mL) and the MIC of ASA was 100 μM (117 µg/mL), suggesting that TE1 and ASA were superior to the saponin mixture (MIC of 125 µg/mL, MFC of 250 µg/mL) (Table 1). The higher MIC of the mixture might be due to the presence of compounds without antifungal activity in the mixture, resulting in a decrease in the ratio of antifungal substances. Proteins, fats, sugars, and phenolic substances were possible residues in the purified tea saponin mixture [42]. Polysaccharides and phenols have been reported to have antifungal activity, but the biological activity of monomer components still has limitations [43,44]. The MIC of the phenolic mixture isolated from tea seeds for *C. albicans* was greater than 2 mg/mL. Interactions between substances might also affect antifungal activity. Numerous studies have reported that polysaccharides benefited the repair of damaged cell membranes [45,46,47]. The existence of tea seed polysaccharides might also have an antagonistic effect on saponins, resulting in a weakened antifungal effect. Further studies are needed to prove this.

The kill curve showed that the fungicidal efficiency of ASA was higher than that of TE1. According to previous reports, the biological activities of triterpenoid saponins with different structures were varied. Some modification groups, such as the type of sugar group on the C3 position, had a crucial impact on the cytotoxicity, hemolysis [48], and antifungal activity [49]. Acetyl groups in the C21 and C22 positions increased antihyperlipidemic activity [50]. A cinnamoyl group in the C22 position increased the cytotoxicity, while an acyl group in the C22 position decreased the cytotoxicity [51]. The groups in the C22 position of ASA and TE1 were an angelic acid moiety and an acetyl group, respectively. Accordingly, ASA had better antifungal activity than TE1, implying that the group type in the C22 position might also have an impact on the antifungal activity. Other studies reported that saponins damaged the integrity of membranes by binding their hydrophobic parts to cholesterol [25,26,52,53]. Therefore, the hydrophobicity of saponins may also affect their antifungal activity. Studies on the structure–activity relationship and molecular targets of saponins are needed to further explore their potential as antifungal agents.

Cell membranes have important physiological functions, including maintaining the stability of the intracellular environment, signal transduction, and material transportation [54]. The integrity of cell membranes is crucial for cell viability, and membrane damage can lead to high cytotoxicity. Numerous studies have shown that the cell membrane of fungi is a target for inhibiting fungal growth and reproduction [55]. In this study, it was found that saponins caused significant damage to cell membranes, which is consistent with previous studies [16,56]. Morphological changes in cell membrane damage were observed using SEM and TEM. The damaged cell membrane integrity was also verified by the PI staining and leakage experiments.

Ergosterol is an important component of the fungal cell membrane, which is of great significance for maintaining normal physiological functions [57]. Ergosterol biosynthesis is the most important cellular pathway targeted by antifungal compounds [58,59]. Ergosterol is synthesized on the endoplasmic reticulum by 25 enzymes in sequence. The three major classes of clinical antifungal substances include azoles, allylamines, and morpholines. Their targets are the *EGR11* gene encoding 14α-demethylase, the *ERG1* gene encoding squalene epoxidase, and the *ERG2* gene encoding sterol C8 isomerase, respectively. By interfering with the biosynthesis of ergosterol, they damage the cell membrane. Through transcriptome analysis, it was found that after saponin treatment, genes related to the ergosterol biosynthesis pathway in *C. albicans* cells, including *ERG1*, *ERG2*, *ERG3*, *ERG5*, *ERG11*, *ERG24*, *ERG26*, and *ERG251*, were downregulated, indicating that saponins inhibited ergosterol synthesis.

Previous studies demonstrated that the higher content of ergosterol was related to the higher resistance of the strain [60,61]. Jiang et al. found that the average ergosterol content in the resistant group was higher than that in the susceptible group [62]. Differences in sterol structures in cell membranes also affected the effectiveness of antifungal agents. Amphotericin B, a polyene antifungal active substance, has different recognition effects on ergosterol and cholesterol. The combination of amphotericin B and ergosterol mainly highlights the role of the cyclomethyl group at the C19 position on the sterol ring structure and also highlights the binding force of the chain double bond without affecting the methyl group at the C21 position. On the contrary, binding with cholesterol does not affect the C19 methyl group on the ring; rather, it comes into contact with the C26 and C27 methyl groups at the end of the chain [63]. Binding with sterols was one of the ways for saponins to inhibit fungi [64]. In this study, the ergosterol binding activity of the two saponins was verified. Yu et al. found that the MIC of the saponin mixture was 64 µg/mL for the *C. albicans* YEM30 strain and 78 µg/mL for the *C. albicans* CMCC98001 strain [56], indicating that the antifungal activity of saponins might be also related to the content and type of sterols on the cell membrane of the strain. The relationship between the structure of the saponins and the content and structure of the sterols in the cell membrane of the fungal strains still deserves further research.

In addition to targeting ergosterol, ROS induction plays an important role in the potential mechanisms of antifungal drugs. The overproduction of ROS may lead to cell damage and death [65]. The presence of ROS causes a decrease in mitochondrial membrane potentials, which had previously been used to determine mitochondrial function [66]. Previous studies have mostly elucidated the antifungal mechanism of saponins against *C. albicans* strains from the perspective of ROS. Li et al. found that the saponins extracted from *Camellia Oleifera* seeds affect mitochondrial dysfunction by inducing the production of reactive oxygen species, leading to the death of *C. albicans* [18]. In this study, we also investigated the role of oxidative stress pathways against *C. albicans* (Figure 4). Our results indicated that saponins caused a significant elevation in intracellular ROS levels, and a significant decrease in the mitochondrial membrane potential, which are negatively correlated. Therefore, the death of *C. albicans* may be caused by mitochondrial dysfunction, in turn caused by the accumulation of ROS through the depolarization of the mitochondrial membrane potential. This is concordant with prior studies [18]. However, supplementation with antioxidants (NAC and VC) did not decrease the level of ROS and did not restore *C. albicans*’ damage potential, which differs from the previous studies. The above results indicate that the oxidative stress pathway was not the main pathway for saponins leading to *C. albicans*’ death.

## 4. Materials and Methods

### 4.1. Strains and Chemicals

The strain *C. albicans* (ATCC 10231) was obtained from the Guangdong Microbial Culture Collection Center (Guangzhou, China) and grown on yeast extract–peptone–dextrose agar (YPD, Sangon Biotech (Shanghai) Co., Ltd., Shanghai, China). Before each test, inoculations of single colonies of the strain were cultured in YPD liquid media and incubated overnight at 30 °C at 200 rpm.

Amphotericin B (AMB), caspofungin (CPF), fluconazole (FLC), ergosterol (Shanghai Aladdin Biochemical Technology Co., Ltd., Shanghai, China), and sorbitol (Sigma-Aldrich Co., St. Louis, MO, USA) were used in this study.

### 4.2. Preparation of TE1 and ASA

*Camellia sinensis* seed cake was provided by the Jinhua Academy of Agricultural Science (Jinhua, China). The tea seed cake (1 kg) was crushed into powder and extracted three times with 70% MeOH (10 L) at 65 °C under reflux each for 2 h. After the removal of the solvent from the MeOH solution under reduced pressure, it was then extracted successively with hexane, ethyl acetate, and saturated n-butanol [67]. The n-butanol fraction was purified using a D101 macroporous resin absorption, and the saponin mixture was prepared by the collection and concentration of 70% ethanol elution [67]. The saponin monomers TE1 and ASA (Figure 1) were purified via a Waters Auto Purification system (2545 pump-PCM 510 Compensated pump-2767 sample manager-2489 DUV-2424 ELSD, Waters, Milford, MA, USA). For complete separation, purification, and characterization data, see Supplementary Methods.

### 4.3. Antifungal Susceptibility Tests

Minimum inhibitory concentrations (MICs) were determined according to the broth microdilution method of the Clinical Laboratory Standards Institute (CLSI), with slight modification [68]. Cells were diluted to a concentration of 1 × 10^6^ CFU/mL in a YPD medium and then mixed together with different concentrations of TE1 and ASA. Then, 200 μL of solutions was added to 96-well plates (Corning Int., Corning, NY, USA) containing 3.125–400 μM of TE1 and ASA, respectively, and incubated for 24 h at 30 °C. AMB (0.125–16 μg/mL), FLC (15.625–2000 μg/mL), and CFP (0.25–8 μg/mL) were used as positive controls. DMSO and distilled water were used as negative, solvent, and growth control, respectively. Culture medium was used as a blank control. MIC was set for wells without turbidity. In addition, the MIC wells and the wells with concentrations above the MIC were plated onto YPD agar plates with 10 μL per well and incubated overnight at 30 °C. The minimum fungicidal concentration (MFC) was defined as the lowest concentration where no colony growth was observed. Each experiment was performed in triplicate.

### 4.4. Time–Kill Curves

The kill curve was used to investigate the fungicidal effects of TE1 and ASA. The YPD medium of fresh *C. albicans* cells (1 × 10^6^ CFU/mL) was combined with 0, 1/2 MIC, 1 MIC, and 2 MIC of TE1 and ASA, respectively. Cells were incubated at 30 °C, 200 rpm for 48 h and sampled at different time points (0 h, 1 h, 2 h, 3 h, 4 h, 5 h, 10 h, 24 h, and 48 h). Following these time periods, we took an aliquot of 10 μL and serially diluted it with sterile PBS, and then plated it onto YPD agar plates. These were incubated for 24 h at 30 °C, after which the CFUs were counted. Curves were constructed by plotting the log10 of CFU/mL against time. Results are reported as the means of three separate experiments.

### 4.5. Live/Dead Fungicidal Fluorescent Imaging

Further detailed evaluation of the antifungal effects of TE1 and ASA was performed using live/dead fungicidal fluorescent imaging of confocal laser scanning microscopy (CLSM, FV1200, Olympus, Tokyo, Japan). Briefly, cells (1 × 10^7^ CFU/mL) were treated with 1 MIC of TE1 and ASA at 30 °C, 200 rpm, for 2 h. Next, the cells were washed twice with sterile PBS and incubated with 2 μg/mL PI (Solarbio Science & Technology Co., Ltd., Beijing, China) and 5 μM SYTO-9 (Invitrogen Detection Technologies, Eugene, OR, USA) at 37 °C in darkness for 15 min. After being stained, samples were washed and resuspended in PBS. Finally, the samples were put on a glass cover slip and imaged by CLSM.

### 4.6. Morphology Observation of C. albicans ATCC 10231

Cells were treated with 1 MIC of TE1 and ASA at 30 °C, 200 rpm for 2 h. The ultrastructure observation of the treated *C. albicans* cells was achieved by Shiyanjia Lab on a scanning electron microscopy (SEM) and transmission electron microscope (TEM) according to the standard protocols.

### 4.7. Cellular Leakage Effect

When the fungal membrane was damaged, the nucleic acids and proteins leaking from the fungal culture medium could be measured. Briefly, after treatment with different concentrations (0, 1/4 MIC, 1/2 MIC, and 1 MIC) of TE1 and ASA, the *C. albicans* cell cultures were centrifuged for 10 min at 5000 rpm. Then, the suspensions were detected by a Synergy H1 microplate reader (BioTek Instruments, Inc., Winooski, VT, USA) at OD260 and OD280.

### 4.8. Sorbitol Protection Assay

The experimental method was conducted according to the literature reports [23,24]. The effect of TE1 and ASA on the fungal cell wall was conducted by sorbitol protection assay. The specific step was that the 0.8 M sorbitol in the absence and presence of YPD medium, and the MIC of TE1 and ASA, were determined using the broth microdilution method after 2 and 7 days. Caspofungin was used as a positive control.

### 4.9. Ergosterol Binding Assay

The experimental method to evaluate whether the compound binds to fungal membrane sterols through ergosterol binding experiments was conducted according to the literature reports [23,24]. In brief, with the addition of 0, 50, 100, 150, 200, and 250 μg/mL ergosterol in YPD medium, the MIC value of TE1 and ASA was determined after 48 h in accordance with the broth microdilution method described above. Amphotericin B was used as a positive control.

### 4.10. Reactive Oxygen Species and Membrane Potential Assays

ROS were detected by the DCFH-DA assay (Beyotime Biotech, Shanghai, China). MMP was detected by the Mitochondrial Membrane Potential Assay Kit with JC-1 (Beyotime Biotech, Shanghai, China). The *C. albicans* cells (1 × 10^7^ CFU/mL) were cultured with various concentrations of TE1 and ASA (0, 1/4 MIC, 1/2 MIC, and 1 MIC) for 2 h at 30 °C. Then, the treated cells were washed twice with PBS and stained with DCFH-DA or JC-1 for 30 min in the dark. Subsequently, the cells were washed with PBS and resuspended in PBS post-centrifugation. Finally, the absorbance was measured using a Synergy H1 microplate reader. CCK-8 assay (Beyotime Biotech, Shanghai, China) was used to measure viable cell numbers. The percentage of control was used to express ROS. The MMP was determined as the ratio of JC-1 monomer (490/530 nm) and JC-1 aggregate (525/590 nm) fluorescence intensity. All experiments were performed in triplicate.

### 4.11. The Effect of the Antioxidant on the Fungicidal Activity of Saponins

The *C. albicans* cells (1 × 10^7^ CFU/mL) were pretreated with or without antioxidants, including NAC (5 mM and 10 mM) and VC (5 mM and 10 mM) at 30 °C for 1 h, respectively. Then, they were cultured with 1 MIC of TE1 and ASA for 2 h at 30 °C, respectively. The detection method of ROS was the same as in Section 4.10.

### 4.12. Transcriptome Analysis

The Yeast RNA Extraction Kit (Aidlab Biotech, Beijing, China) was used for total RNA extraction. The *C. albicans* cells (1 × 10^7^ CFU/mL) were cultured with 1 MIC of TE1 and ASA for 2 h at 30 °C, with three independent biological replicates. The RNA concentration and purity were verified. The cDNA libraries were constructed with an Illumina NovaSeq 6000 platform by Personalbio Technology (Shanghai, China) with paired-end reads. The clean reads were mapped to the *C. albicans* SC5314 genome sequence with HISAT2. The analysis of the differential expressed genes (DEGs) was performed by DESeq2 software (v1.40.1) between the TE1 treatment [69], the ASA treatment, and the control, and |log2foldchang| > 1 and *p*-value < 0.05 were used as conditions for screening DEGs. On the basis of the GO and KEGG pathways, functional annotation was performed using the GO (http://geneontology.org, accessed on 8 December 2022) and KEGG (http://www.genome.ad.jp/kegg/, accessed on 14 December 2022) databases [70].

### 4.13. Real-Time Quantitative Reverse Transcription PCR (qRT-PCR) Validation

Sixteen DEGs were validated by qRT-PCR with the paired primers (Table S3), with 18S rRNA as the reference gene. Total RNA was reverse transcribed to cDNA using SYBR^®^ Premix Ex Taq™ (Monad Biotech, Shanghai, China). The PCR program was as follows: 95 °C for 10 min, followed by 40 cycles of 95 °C for 10 s, 60 °C for 10 s, and 72 °C for 15 s. The relative expression levels of the genes were normalized using the 2^−ΔΔCT^ method.

### 4.14. Statistical Analysis

All data are presented as the mean ± standard deviation (three replicates). The results were analyzed with SPSS Version 22.0 using one-way ANOVA to demonstrate the significant differences (*p* < 0.05, *p* < 0.01). GraphPad Prism (v9.4.1) was used to process the data and generate the figures.

## 5. Conclusions

In this study, the fungicidal effects of saponin monomers isolated and purified from *Camellia sinensis* seeds, which were TE1 and ASA, were highlighted against fluconazole-resistant *Candida albicans* strains for the first time, and there were differences in antifungal activity between the monomers. TE1 and ASA bound with ergosterol on the fungal cell membrane to increase the cell membrane permeability and disrupted the cell membrane integrity of the *C. albicans* cells. Moreover, the accumulation of intracellular ROS treated with TE1 and ASA lead to mitochondrial dysfunction, affecting energy metabolism, but this may not be the main cause of *C. albicans*’ death. These results suggest that tea seed saponins could be used as broad-spectrum and potential fungicides for the management of pathogenic fungi with ergosterol as the main target. In addition, the structure–activity relationship of the antifungal activity of saponins remains to be further studied.

## Figures and Tables

**Table 1:** Antifungal activity against *C. albicans* ATCC 10231.

Compounds	MIC	MFC
AMB	0.5 μg/mL	0.5 μg/mL
CPF	0.25 μg/mL	0.5 μg/mL
FLC	>2000 μg/mL	>2000 μg/mL
Saponin mixture (purity > 96%)	125 μg/mL	250 μg/mL
TE1	100 μM	100 μM
ASA	100 μM	100 μM

AMB, amphotericin B; CPF, caspofungin; FLC, fluconazole; TE1, theasaponin E1; ASA, assamsaponin A.

**Table 2:** MIC values of TE1, ASA and CPF against *C. albicans* ATCC 10231.

MIC TE1				MIC ASA				MIC CPF			
Day 2		Day 7		Day 2		Day 7		Day 2		Day 7	
(-) S	(+) S	(-) S	(+) S	(-) S	(+) S	(-) S	(+) S	(-) S	(+) S	(-) S	(+) S
100 μM	100 μM	100 μM	100 μM	100 μM	100 μM	100 μM	100 μM	0.25 μg/mL	8 μg/mL	0.5 μg/mL	8 μg/mL

TE1, theasaponin E1; ASA, assamsaponin A; S, sorbitol; (+), presence; (-) absence.

**Table 3:** MIC values of TE1, ASA and AMB against *C. albicans* ATCC 10231.

Compounds	Ergosterol Concentration (μg/mL)					
	0	50	100	150	200	250
TE1	100 μM	100 μM	100 μM	100 μM	100 μM	200 μM
ASA	100 μM	100 μM	100 μM	100 μM	100 μM	200 μM
AMB	0.5 μg/mL	2 μg/mL	4 μg/mL	8 μg/mL	16 μg/mL	32 μg/mL

TE1, theasaponin E1; ASA, assamsaponin A; AMB, amphotericin B.

## Data Availability

The authors declare that all data generated or analyzed during this study are included in this published article and its Supplementary Information files. Related data are available from the authors upon reasonable request.

## Supplementary Materials

The supporting information can be downloaded at: https://www.mdpi.com/article/10.3390/ijms24119350/s1.
