# Supplementary material for: Anti-Candida albicans Effects and Mechanisms of Theasaponin E1 and Assamsaponin A
Source: Int J Mol Sci. 2023 May 27;24(11):9350. doi: 10.3390/ijms24119350 (PMC10253373; doi:10.3390/ijms24119350)
Supplement: Supplementary file 1 [file ijms-24-09350-s001.zip › ijms-2362007-supplementary.pdf]

Supplementary Materials for

Anti-*Candida albicans* Effects and Mechanisms of Theasaponin E1 and Assamsaponin A

Supplementary Tables

Supplementary Table S1. Transcriptome data quality control.

Sample	Raw Read Number	Raw Bases	Raw Q30 Number	Raw N Rate	Raw Q20 Rate	Raw Q30 Rate
Control-1	41786928	6309826128	5850599592	0.000788	97.47	92.72
Control-1	47489568	7170924768	6687522569	0.00093	97.71	93.25
Control-3	38251358	5775955058	5404483320	0.000795	97.84	93.56
TE1-1	38021816	5741294216	5317132883	0.000789	97.38	92.61
TE1-2	39392002	5948192302	5550805261	0.000789	97.69	93.31
TE1-3	45244014	6831846114	6338555973	0.000787	97.46	92.77
ASA-1	43608994	6584958094	6118634651	0.000791	97.54	92.91
ASA-2	39230128	5923749328	5504170948	0.000796	97.52	92.91
ASA-3	44734742	6754946042	6266585664	0.000796	97.47	92.77

Supplementary Table S2. Statistics on the number of differentially expressed genes in different sectors.

Comparison	All	Up	Down
Control vs. TE1	2135	1344	791
Control vs. ASA	2105	1339	766
TE1 vs. ASA	17	15	2

The criteria for screening differences were DESeq2 |log2foldchang| > 1 and p-value < 0.05.

Supplementary Table S3. Primers for qRT-PCR.

**Primer**	**Sequence**
18S rRNA-F	AATTACCCAATCCCGACAC
18S rRNA-R	TGCAACAACTTTAATATACGC
ERG1-F	ACCGGCTGGTATCAAGGCATT
ERG1-R	GGCATCAGGAACTGGCTTCAC
ERG3-F	TGCTTCTCATGCTTTCCATCCA
ERG3-R	GGACAGTGTGACAAGCGGTAC
ERG11-F	TGGAGACGTGATGCTGCTCAA
ERG11-R	GCAGAAGTATGTTGACCACCCA
ERG24-F	GGTGACTTAGCGTGGGTTCCT
ERG24-R	CACCAGCCTTCCACCAACAATT
ERG26-F	CAGCTTCACCGATGCATGGATT
ERG26-R	CCGCAGGACGTAAACAAACAGT
ADH2-F	CATTGGTTGGTGGTCACGAAGG
ADH2-R	TGAACAGCGTCAGCAGTAGCA
ADH3-F	GCTGCTGTTGCTACTGATTCTG
ADH3-R	CCAATTTCTCAACCGCACCTTT
ALD5-F	GCACAAGCCGCATACCACAA
ALD5-R	CCAACCACCACAGGATCTGAAG
ALD6-F	TGCTGATGCTCAAGGTGATGTT
ALD6-R	GCTCCAGGTACACGTTCACTTG
ADH1-F	GCTACTGCTGATGCTGTCCAAG
ADH1-R	TCGTCACCACCGTCAATAGCA
CDC19-F	ACCTACAACCCAAGACCAACCA
CDC19-R	AACAGCGGCAACAGCACAAG
CHO2-F	GTCGGGTTCGGATTCTCCTTCT
CHO2-R	CCCATAACGCCACAAACTCCAA
GUT2-F	TAGTGCTGCTGTTGGTGGTACA
GUT2-R	ACGAGATGGTGGTGGTGGAAT
ENA21-F	TTCTGTGGTGCCGATGACGATA
ENA21-R	ACGAGCAAGTATCAGTGTTGGC
PHO88-F	TGGTGTTCCAGCTTCTGGTGAT
PHO88-R	CGGCAGTTTCAGCACTTTCAAC
CDR1-F	ACGATCCAACACCAGGGAAACT
CDR1-R	TCTCGCAACACCATACCTCACT

Supplementary Methods

The high-purity saponin mixture purified by macroporous resin enters the preparation liquid phase for further separation and purification.

Preparation and isolation of differentiated samples from the tea saponin mixture

Differentiated samples purified via Waters Auto Purification system (2545 pump-PCM 510 Compensated pump-2767 sample manager-2489 DUV-2424 ELSD, Waters, Milford, Massachusetts, USA), fitted with a Waters Sunfire C18 column (5 μm, 30 × 250 mm, Waters, Milford, MA, USA). The injection volume was 1800 μL, and the mobile phase flow rate was 30 mL/min. Solvent System A: Water contains 0.1% formic acid; B: CH3CN / CH3OH = 1 / 1 (containing 0.1% formic acid). The mobile phase elution gradient was 5% B at 0.01 min, ramped linearly to 40% B at 3 min, 70% B at 80 min, 95% B at 90 min, then back to 5% B at 100 min. This preparation procedure yields three main peaks (67.86 min of peak1, 71.37 min of peak2, and 76.25 min of peak3).

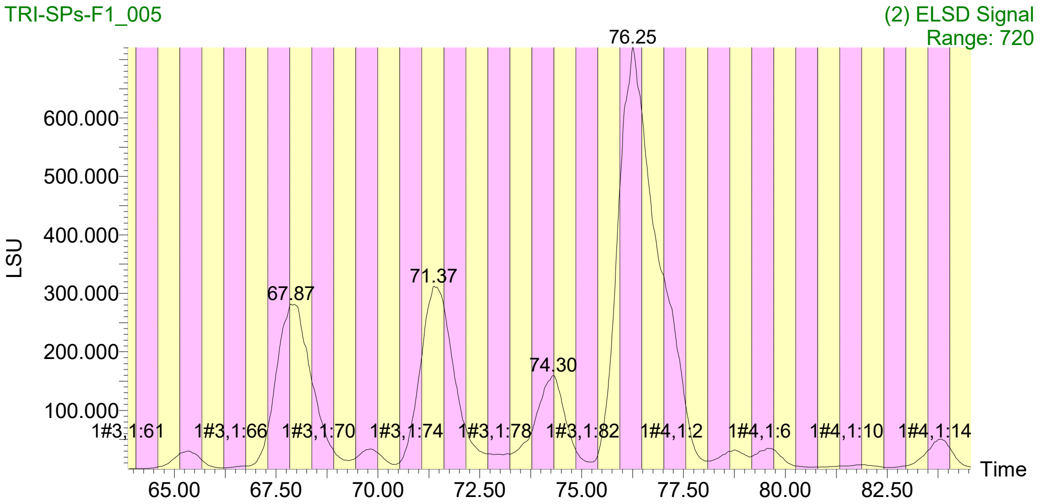

Further purification

Further separate the substances of the three main peaks via YMC-ODS-A column (5 μm, 30 × 150 mm). The mobile phase elution gradient was 32% B at 0.01-3 min, ramped linearly to 43% B at 30 min, 95% B at 35 min, then back to 32% B at 40 min for isolating 2 substances from peak1.

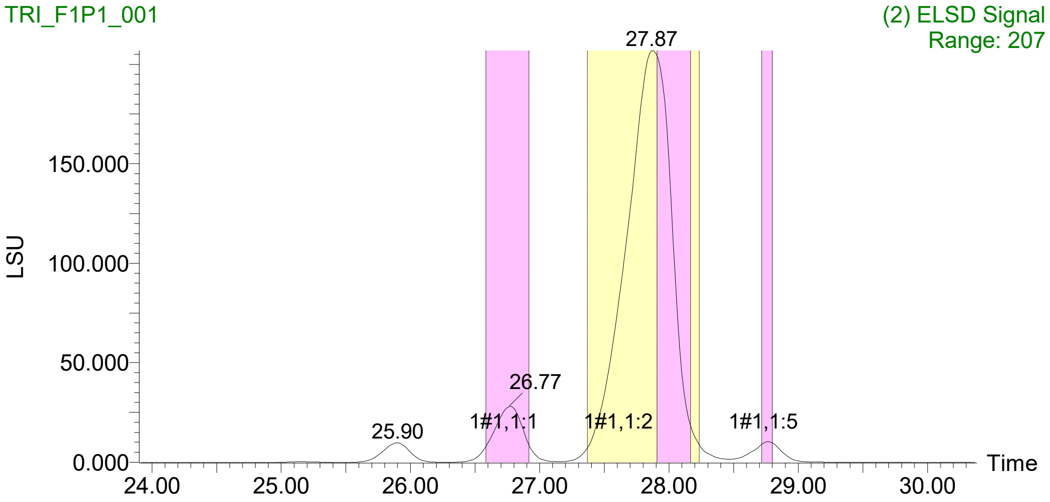

The mobile phase elution gradient was 31% B at 0.01-3 min, ramped linearly to 44% B at 30 min, 95% B at 35 min, then back to 31% B at 40 min for isolating 2 substances from peak2.

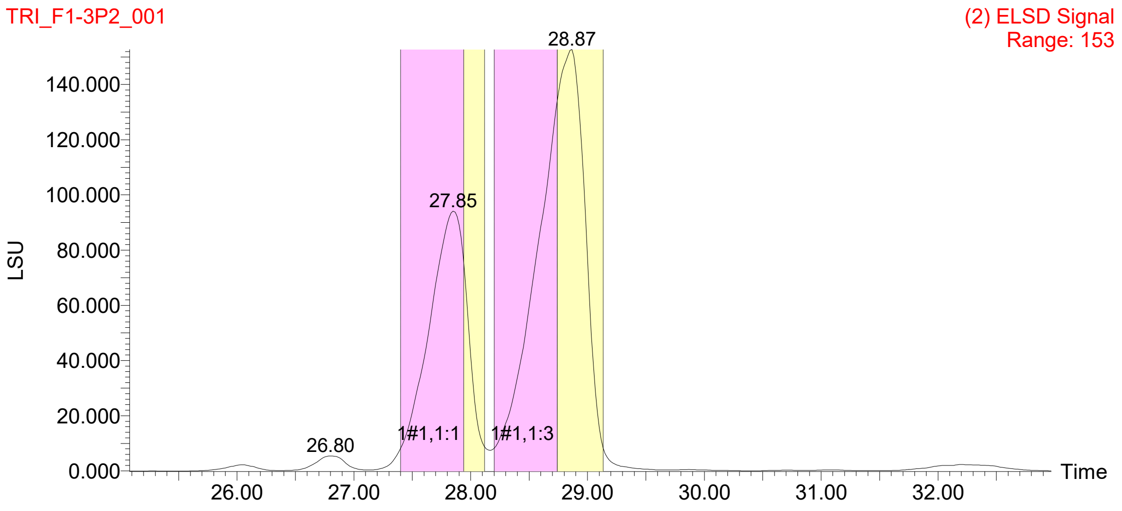

The mobile phase elution gradient was 32% B at 0.01-3 min, ramped linearly to 45% B at 30 min, 95% B at 35 min, then back to 32% B at 40 min for isolating 3 substances from peak3.

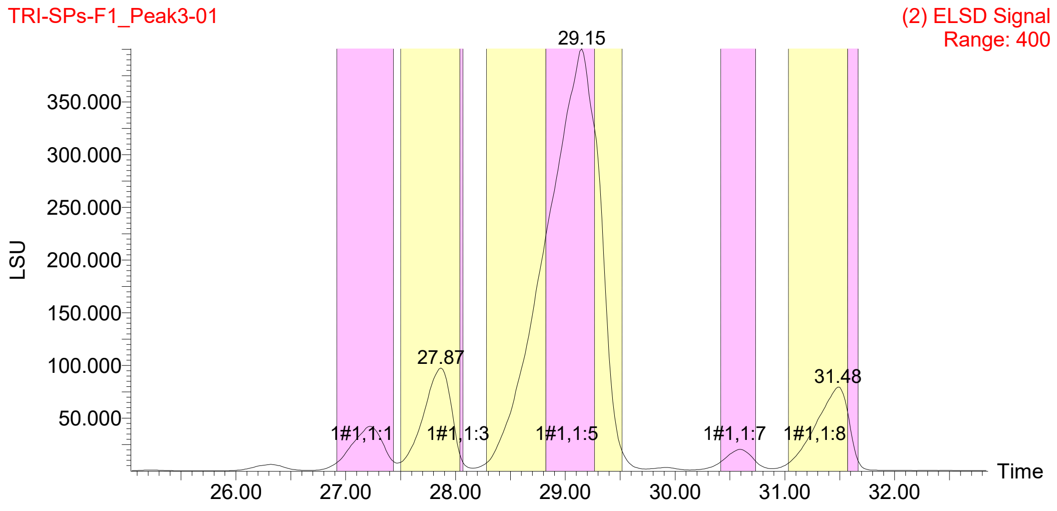

Characterization data of Theasaponin E1 and Assamsaponin A

Product Name: **Theasaponin E1**, Exact Mass: 1230.5669

CNMR

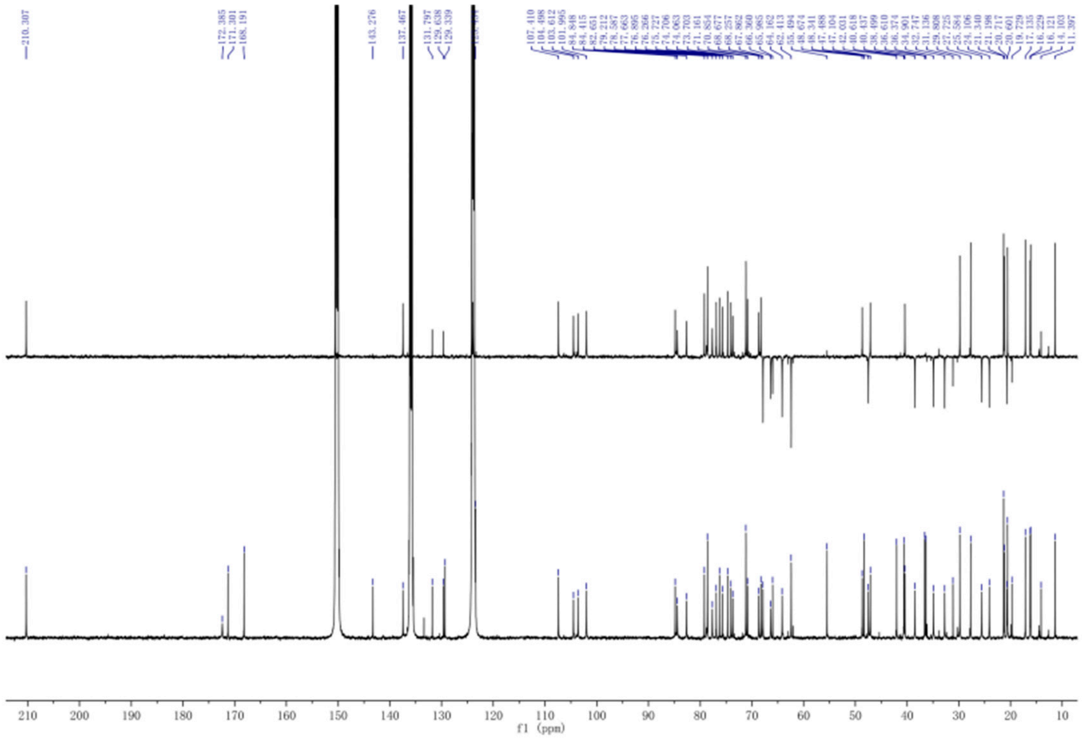

HNMR

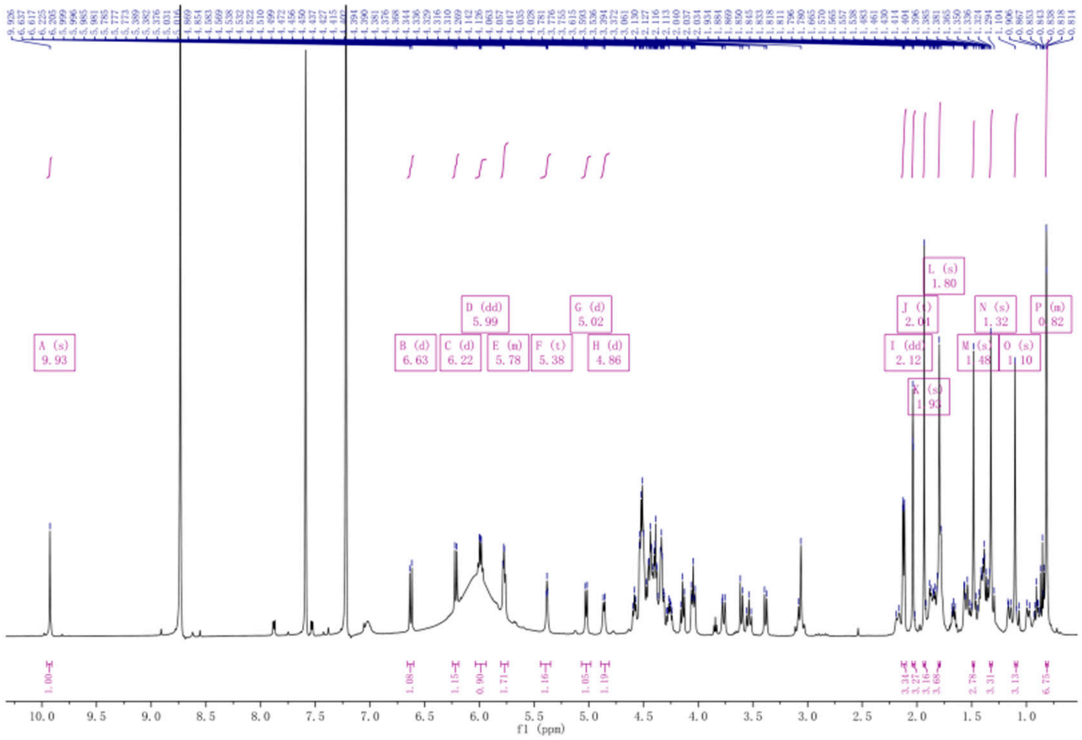

LC-MS

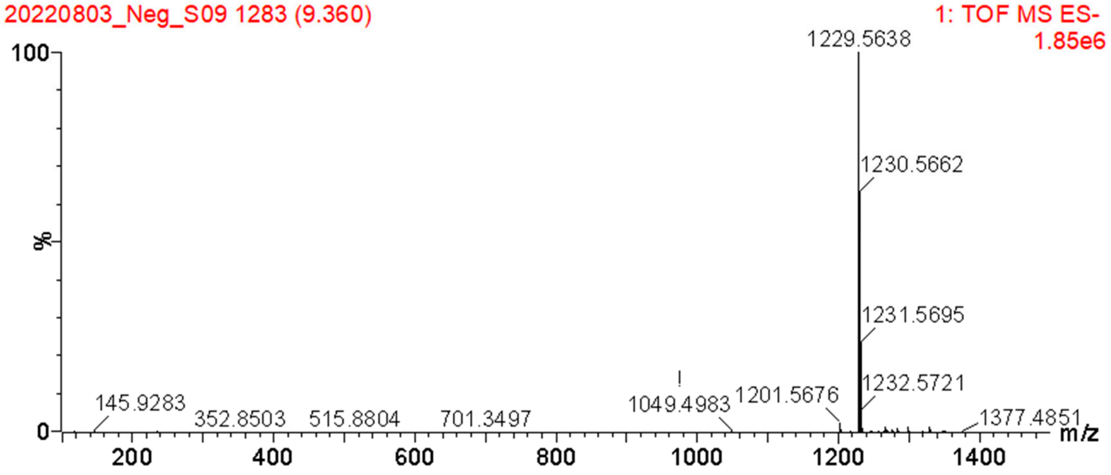

Purity

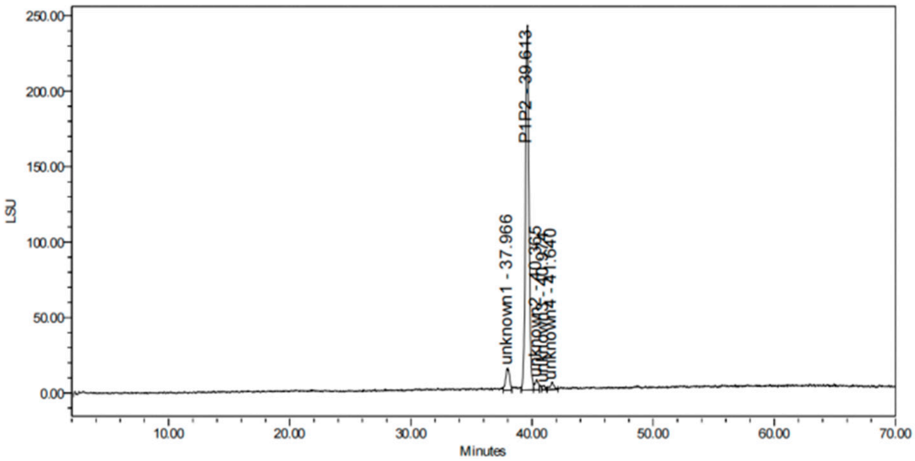

	Peak Name	RT	Area	% Area	Height
1	unknown1	37.966	325567	5.93	14512
2	P1P2	39.613	4871574	88.66	241822
3	unknown2	40.365	134027	2.44	6614
4	unknown3	40.974	52993	0.96	2747
5	unknown4	41.640	110426	2.01	4787

Product Name: **Assamsaponin A**, Exact Mass: 1172.5615

CNMR

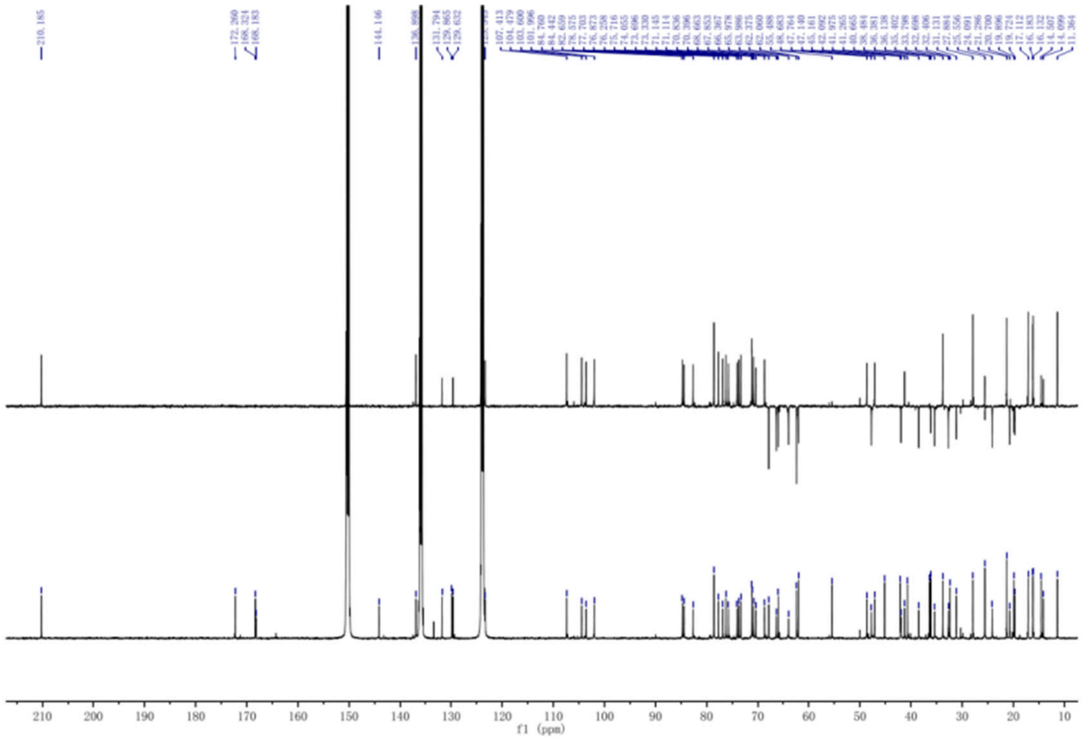

HNMR

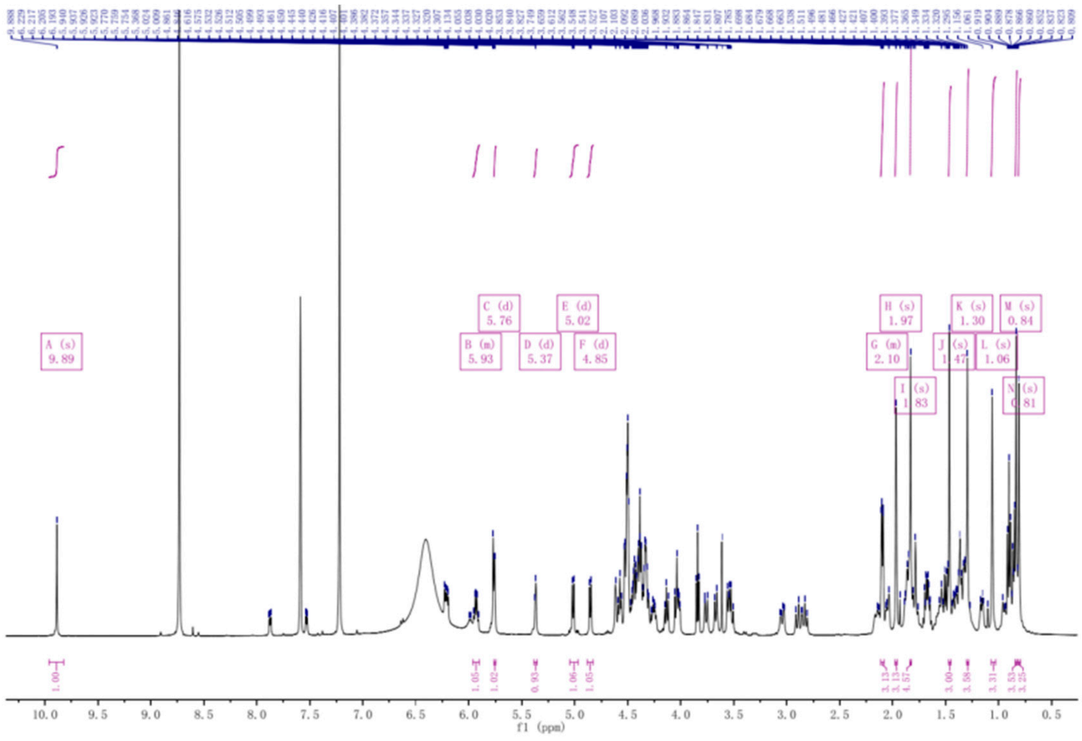

LC-MS

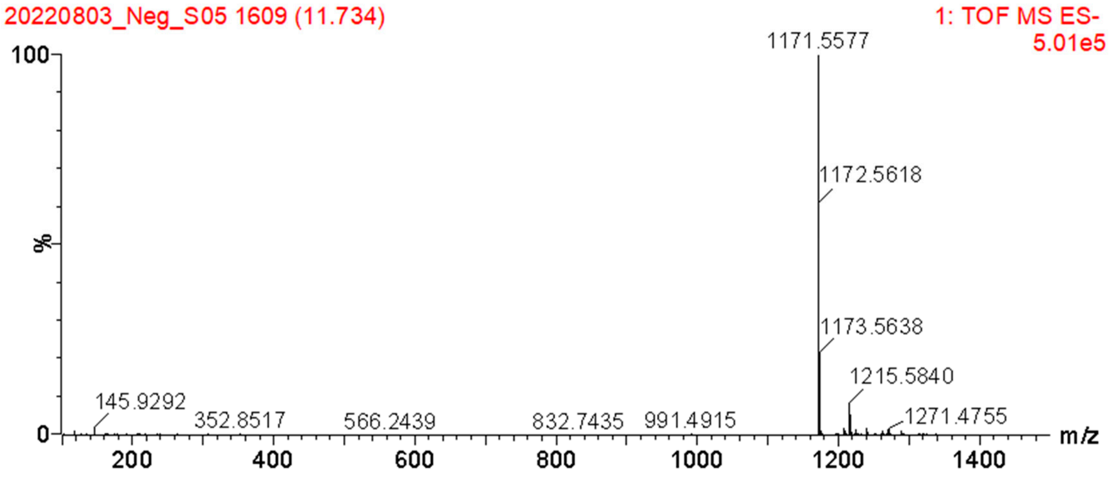

Purity

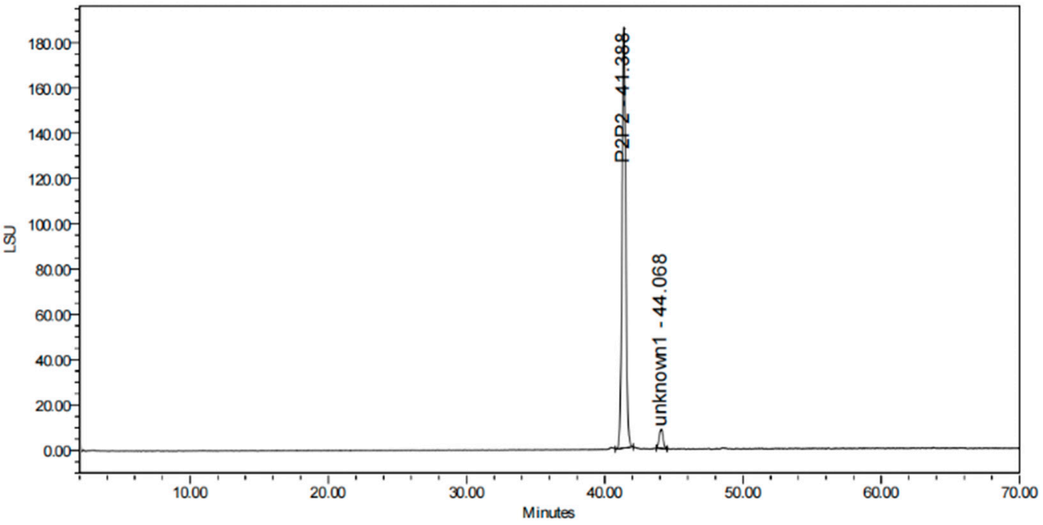

	Peak Name	RT	Area	% Area	Height
1	P2P2	41.388	3446659	95.40	185882
2	unknown1	44.068	166375	4.60	8407
